# Membrane Heterogeneity Beyond the Plasma Membrane

**DOI:** 10.3389/fcell.2020.580814

**Published:** 2020-11-19

**Authors:** Hong-Yin Wang, Deepti Bharti, Ilya Levental

**Affiliations:** ^1^Department of Molecular Physiology and Biological Physics, University of Virginia, Charlottesville, VA, United States; ^2^National Institute of Technology, Rourkela, India

**Keywords:** membrane domain, lipid raft, organelle, Golgi, endoplasmic reticulum

## Abstract

The structure and organization of cellular membranes have received intense interest, particularly in investigations of the raft hypothesis. The vast majority of these investigations have focused on the plasma membrane of mammalian cells, yielding significant progress in understanding membrane heterogeneity in terms of lipid composition, molecular structure, dynamic regulation, and functional relevance. In contrast, investigations on lipid organization in other membrane systems have been comparatively scarce, despite the likely relevance of membrane domains in these contexts. In this review, we summarize recent observations on lipid organization in organellar membranes, including endoplasmic reticulum, Golgi, endo-lysosomes, lipid droplets, and secreted membranes like lung surfactant, milk fat globule membranes, and viral membranes. Across these non-plasma membrane systems, it seems that the biophysical principles underlying lipid self-organization contribute to lateral domains.

## Introduction

The exquisite complexity and capability of biology are enabled largely by physical and/or biochemical compartmentalization. A predominant mechanism for biological compartmentalization is provided by lipid membranes, which partition intracellular space into membrane-bound organelles. Further, these membranes themselves can be compartmentalized into functional, lateral subdomains (Garcia-Parajo et al., 2014). The most well-studied example of such membrane subdomains is the so-called membrane rafts. In the present manuscript, “raft” describes lipid-driven, relatively ordered assemblies of lipids and proteins typically associated with the mammalian plasma membrane (PM) (Lingwood and Simons, 2010). Such domains result from preferential associations between cholesterol, sphingolipids, and other saturated lipids that produce a tightly packed lipid environment different from, and coexisting with, less tightly packed, non-raft membrane regions (Levental I. et al., 2020). In living cells, these effects are believed to produce nanoscopic, dynamic domains whose specific properties (sizes, lifetimes) are highly context-dependent. These domains have been implicated in various physiological roles, most notably signaling (Simons and Toomre, 2000) and membrane trafficking (Simons and Ikonen, 1997; Schuck and Simons, 2004), although their general function can be summarized as lateral sorting of proteins, which could facilitate or inhibit their interactions with other biomolecules (Lorent et al., 2017). There is also a growing body of evidence supporting similar membrane domains in prokaryotes, which may depend on sterol-analogous molecules like hopanoids (Saenz et al., 2012; Saenz et al., 2015; Belin et al., 2018) or exogenous sterols scavenged from other organisms (Huang and London, 2016). This work has been expertly reviewed elsewhere (Lopez and Koch, 2017) and will not be discussed in detail here.

The raft phenomenon has been studied extensively, with much of that attention focused on functional domains of the PM. The reasons for this inordinate attention to PM rafts are both historical and practical. The raft concept was originally proposed to explain the unique sterol- and sphingolipid-enriched composition of the apical PM in epithelial cells (van Meer et al., 1987). In this context, self-assembly of raft-preferring lipids and proteins would aid in the sorting of PM-destined components in the trans-Golgi apparatus. Thus, a reasonable assumption would be that the PM is raft-rich. Functionally, the PM is the site for exchanging material and signals between cells and extracellular environments; thus, many interesting and important biological events are involved. Compositionally, the PM contains high levels of cholesterol (up to 40% mol; Levental et al., 2016, 2017; Lorent et al., 2020) and sphingolipids (10%; Symons et al., 2020), which tend to promote the formation of ordered raft domains (Jacobson et al., 2007). Finally and perhaps most significantly, the PM is large, relatively flat, and easily accessible for external labeling and manipulation, making it the most experimentally convenient membrane of the cell.

Despite these valid reasons for attention on mammalian PM domains, it is possible, if not likely, that similar principles of self-organization can drive membrane domains in other cell membranes. Lipids in other cell membranes have properties that tend to cause favorable interactions between various subclasses, leading to lateral heterogeneity. Examples of such properties include (a) acyl chain saturation, with relatively saturated lipids tending to pack together into ordered solid or liquid domains; (b) cholesterol abundance, with cholesterol interacting preferentially with relatively saturated lipids; (c) headgroup charge, with polyvalent cations or proteins capable of ionic cross-linking (Wang et al., 2002; Yeung et al., 2008; Levental et al., 2009); (d) specific headgroup chemistries driving self-association (Wang and Silvius, 2003; Sodt et al., 2015) and/or lipid-protein interactions (Ewers et al., 2010). Therefore, it is tempting to predict that membrane domains may also be present in organellar membranes beyond the PM.

**FIGURE 1:**
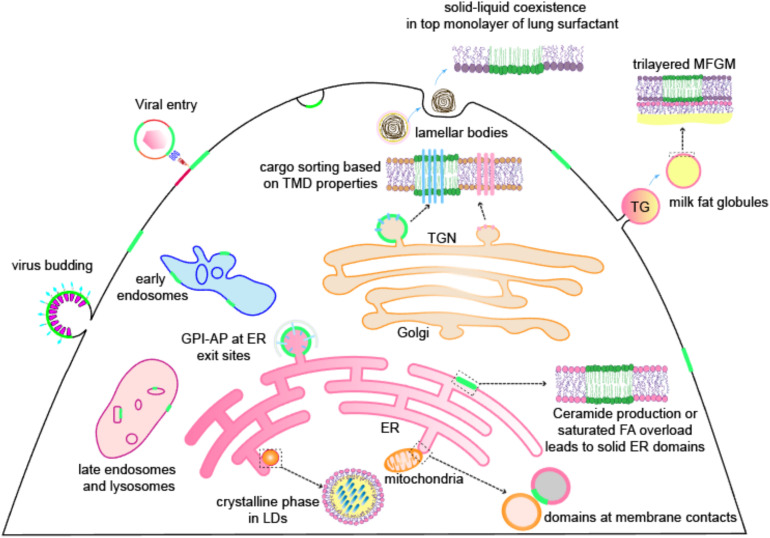
Lipid domains are present on membrane systems, including not only the PM of mammalian cells but also many organellular membranes [endoplasmic reticulum (ER), Golgi, endosomes, lysosomes, lipid droplets (LDs)], viral membranes, and cell-secreted membrane structures like lung surfactants and milk fat globule membranes (MFGMs). Green color represents ordered domains (either liquid or solid). Other abbreviations: TGN, trans-Golgi network; TG, triglyceride; TMD, transmembrane domain; GPI-AP, GPI-anchored protein; FA, fatty acid.

Accumulating evidence indeed strongly suggests, and sometimes directly shows, the presence of lipid domains in various organelles. In this review, we summarize observations of lipid domains found in non-PM membranes, including subcellular organelles [endoplasmic reticulum (ER), Golgi, endosomes, lysosomes, and lipid droplets (LDs)] and non-cellular structures [milk fat and lung surfactant (LS)] (Figure 1). Where applicable, we also discuss the functional relevance of these domains in cellular physiology, with examples from a viral infection of mammalian cells to organelle inheritance in yeast. Although some clues suggest the possibility of lipid domains in nuclear (Cascianelli et al., 2008; Ramamurthy et al., 2018) and mitochondrial membranes (Garofalo et al., 2015), we have not focused on these organelles here. Notably, the lipid domains we reviewed here include not only liquid-ordered (Lo) domains or lipid rafts but also mechanisms of membrane compartmentalization based on solid domains or gel phases. These examples illustrate that the basic biophysical rules of membrane organization are applicable and physiologically relevant across living membrane systems.

## Lipid Domains of the Endoplasmic Reticulum

The ER is the largest cellular membrane system, consisting of a contiguous network of membrane tubes and sheets that comprises the cell’s center for lipid metabolism (Shibata et al., 2006). Although cholesterol is produced in the ER, it is rapidly transported out into other organelles, partly through active machinery present at membrane contact sites (Lev, 2012; Mesmin et al., 2013; Phillips and Voeltz, 2016). For that reason, the ER stores only ∼0.5–1% of the cell’s total cholesterol (Lange et al., 1999), comprising ∼3–5% of all ER lipids (Radhakrishnan et al., 2008). Due to such low cholesterol levels and low levels of saturated sphingolipids, the ER membrane is believed to be mostly disordered (Maxfield and Tabas, 2005; van Meer et al., 2008). Despite this generalization, some observations suggest that functional lipid nanodomains can exist in the ER under certain conditions.

### Lipid Domains at Endoplasmic Reticulum Exit Sites

Membrane protein transport from the ER to the Golgi requires binding to the Sec24 subunit of the coat protein complex II vesicle coat. However, glycosylphosphatidylinositol (GPI)-anchored proteins cannot directly bind to Sec24 because they lack a cytosolic domain. Instead, the P24-P23 protein complex, which can bind to Sec24D, acts as a cargo receptor to mediate the export of GPI-anchored proteins like CD59 or the folate receptor (Bonnon et al., 2010). Because fully processed GPI-anchored proteins typically bear saturated acyl chains (Levental et al., 2010a), there is some suggestion that such GPI-anchor rich exit sites may be related to lipid raft domains on the ER membrane (Bonnon et al., 2010). Consistent with this notion, CD59 and P24-P23 are enriched in detergent-resistant membranes, the biochemical correlate of raft association (Bonnon et al., 2010). Cholesterol depletion disrupts the interaction between CD59 and P24-P23 and thus reduces the export of CD59 to Golgi, whereas the trafficking of non-raft proteins like transferrin receptor and ER–Golgi intermediate compartment-53 is not affected. The results indicate that functional lipid rafts at the ER membrane can assist GPI-anchored proteins in transporting to Golgi.

### Evidence for Lipid Domains at Inter-Organelle Contact Sites

Mitochondria-associate membranes (MAMs) are ER subdomains that play crucial roles in inter-organellar crosstalk between ER and mitochondria required for the homeostasis of both organelles. It has been suggested that these MAMs also have unique lipid compositions that resemble lipid rafts (Garofalo et al., 2016). Indeed, cholesterol and glycosphingolipids appear to be concentrated at the MAMs and potentially required for MAM formation (Sano et al., 2009; Hayashi and Fujimoto, 2010; Garofalo et al., 2016). Such MAM lipid domains may play key functional roles in regulating autophagy, recruiting autophagy-related molecules to the MAM region to facilitate autophagosome formation (Garofalo et al., 2016).

The presence of lipid microdomains at inter-organelle contacts was also recently strikingly supported by observations of large intracellular vesicles induced by hypotonic swelling of adherent cells. This procedure results in the formation of large vesicles derived from various organelles, including ER, mitochondria, endosomes, and lysosomes (although not peroxisomes or LDs) (King et al., 2020). ER-derived vesicles showed microscopically observable phase separation below room temperature, revealed by segregation between disorder-preferring protein Sec61β and order-preferring GPI-anchored protein. Interestingly, these ordered domains appeared solid-like in their physical state, as their proteins were practically immobile as measured by fluorescence recovery after photobleaching (FRAP) (King et al., 2020). Notably, such ordered domains were particularly concentrated at ER contact sites with the PM, endosomes, LDs, and mitochondria, whereas relatively disordered domains formed contacts with lysosomes and peroxisomes. These micron-sized ordered domains at ER-mitochondria contact sites are consistent with the implications of raft nanodomains at ER-MAMs (Garofalo et al., 2016). Altogether, these observations suggest a putative role for ordered membrane microdomains in maintaining contacts responsible for inter-organellar lipid transfer. It is tempting to speculate on the lipid composition of the domains in the organelles in contact with the ER and the possibility that organellar contacts are mediated by apposing ordered domains; the unconventional large intracellular vesicle method may provide a tool to investigate such organization in intracellular membranes (Bag et al., 2020).

### Gel-Like Domains in the Endoplasmic Reticulum

Much of the above discussion has focused on lipid rafts, which are defined in part by being in the Lo state. However, membrane lipids can also form non-liquid ordered domains, most notably the two-dimensional crystalline phase knows as the gel phase, or solid-ordered (So) phase. The So phase is similar to the Lo phase in its high degree of lipid order but distinguished by the negligibly low translational diffusivity of membrane components (van Meer et al., 2008). This state is driven by very tight packing between saturated phospholipids, uninterrupted by sterols. Such functional solid nanodomains have been observed in the ER membrane of budding yeast (Clay et al., 2014). Such domains appear to behave as regulated membrane diffusion barriers for the cortical ER membrane at the neck of a nascent bud, the precursor of the daughter cell in dividing *Saccharomyces cerevisiae*. The function of these diffusion barriers is to retain misfolded or otherwise aberrant proteins in the mother cell, thus protecting the daughter cell from noxious ER stress. These domains are likely composed of sphingolipids, as these are the major saturated lipids in yeast, and inhibition of sphingolipid synthesis pathways (but not other lipids, including glycerolipid, lipid-linked oligosaccharide, and ergosterol) impaired diffusion barrier formation. Because inhibition of ergosterol did not abolish the diffusion barrier, the domains are probably So, rather than Lo. Importantly, such domains can serve as selective barriers to membrane inheritance: the long chains of sphingolipids, especially in an ordered state, present a relatively thick lipid bilayer, which tends to exclude membrane proteins with short transmembrane domains (TMDs) due to hydrophobic mismatch (Clay et al., 2014). This mechanism may be related to aging and renewal of yeast cells, as a perturbation of the diffusion barrier can lead to aberrant inheritance of membrane proteins from mother to daughter cells (Singh et al., 2017) and resulting reduction in culture lifetimes. Although the exact physical state of these diffusion-restrictive domains is not yet clearly defined, lipidomic, and biophysical studies should help resolve the composition and properties of these essential nanodomains.

Consistent with ordered nanodomains in yeast ER, large-scale lipid domains have been observed in mammalian ER under conditions of perturbed metabolism and lipid synthesis (Shen et al., 2017). Feeding HeLa cells with deuterated fatty acids allows observation of the products of lipid metabolism in the ER membrane with minimal perturbation using stimulated Raman scattering microscopy (Shen et al., 2017). Feeding saturated fatty acid increased the synthesis and accumulation of tightly packed saturated lipids, which, without the fluidizing effect of cholesterol, induced large So-like domains in the ER membrane. The domains excluded lipid dyes that partition into fluid membranes and ER membrane proteins and exhibited detergent resistance, low diffusivity, high conformational order, and high domain stability. As expected, large So domains are not compatible with normal cellular physiology and led to cytotoxicity, likely related to induced ER stress (Halbleib et al., 2017). Interestingly, feeding saturated fatty acids, together with their unsaturated counterparts (e.g., the fish oil component docosahexaenoic acid), eliminated solid domain formation and decreased cytotoxicity. These results are consistent with recent findings that maintaining the fluidity of the ER membrane is an essential homeostatic mechanism in mammalian cells (Levental K. R. et al., 2020). Micron-sized protein-free domains were also observed on ER membranes of central nervous system cells from hypothermic ground squirrels during hibernation (Azzam et al., 2000). These domains are reversible after body temperature rises. Whether these domains are related to So domains is worth further investigation.

### Regulation of Membrane Physical State in Endoplasmic Reticulum

The physical state of the ER (e.g., fluidity, lipid packing, membrane thickness, curvature) must be carefully regulated to fulfill its versatile functions. The ER membranes must be fluid and compliant to accommodate nascent membrane proteins with diverse physical properties (Sharpe et al., 2010) but may require local regions to facilitate protein sorting or export (Bonnon et al., 2010; Clay et al., 2014). Perturbations to membrane fluidity can cause deleterious ER stress and result in cytotoxicity (Covino et al., 2016; Ernst et al., 2016; Halbleib et al., 2017; Shen et al., 2017; Levental K. R. et al., 2020). To maintain ER membrane homeostasis, cells use various “sense-and-response” regulators that combine sensitivity to membrane physical properties with a signaling module capable of feedback control (Covino et al., 2018). An archetypal example is Mga2, which sensitively detects aberrant lipid packing density of the ER membrane and responds by upregulating production of the desaturase enzyme that produces unsaturated lipids (Ballweg et al., 2020). Similarly, an overly thick ER membrane can induce the clustering of Ire1, activating the membrane stress response to maintain protein and lipid homeostasis (Halbleib et al., 2017). These and other mechanisms work in parallel to sense and regulate ER membrane biophysical properties and thereby ensure ER membrane homeostasis (Covino et al., 2018).

## Sorting Out the Golgi

Unlike the ER, which is a continuous network with a thin and readily deformable membrane to allow facile insertion of nascent membrane proteins, the Golgi apparatus is a polarized organelle composed of stacked cisternae functionally compartmentalized into cis-, medial-, and trans-Golgi (Boncompain and Weigel, 2018). The major functions of the Golgi complex are to process proteins received from the ER (via posttranslational modifications like glycosylation) and sort them for export to various cellular destinations (PM, endolysosomes, etc.). Due to the diversity of cargos, no single sorting model is likely to explain the trafficking rates and destinations of most membrane-bound proteins. Several different sorting models have been proposed, including selective vesicle trafficking, cisternal maturation, domain partitioning, and others, and it is likely that combinations, or possibly even all of these, are required for optimal Golgi function (Boncompain and Weigel, 2018). Irrespective of the specific sorting models, the importance of lipids and lipid domains in cargo sorting at the trans-Golgi network (TGN) has been well recognized (von Blume and Hausser, 2019). The inositide lipid PI4P is specifically enriched at the TGN (Weixel et al., 2005), where its generation drives cholesterol and ceramide transport from ER to Golgi through lipid transfer proteins oxysterol-binding protein (Mesmin et al., 2013) and ceramide transport protein (Hanada et al., 2003), respectively. Accumulation of ceramide leads to the production of sphingomyelin and other sphingolipids in the Golgi (D’Angelo et al., 2013), particularly the TGN, where their interactions with the accumulating cholesterol may potentiate the formation of raft domains (Simons and Ikonen, 1997; Schuck and Simons, 2004). Another mechanism to enrich rafts in the Golgi is their specific exclusion from Golgi-to-ER retrograde carriers mediated by coat protein complex I (Brugger et al., 2000).

Such Golgi raft domains have been heavily implicated in protein sorting to the PM in non-polarized cells (Yoshimori et al., 1996; Diaz-Rohrer et al., 2014a) and, more specifically, to the apical PM in polarized epithelial cells (Simons and Ikonen, 1997; Paladino et al., 2004; Schuck and Simons, 2004; Surma et al., 2012). In this model, proteins destined for the PM (rich in raft-forming lipids; Gerl et al., 2012; Levental et al., 2016; Symons et al., 2020) are sorted at the TGN via their affinity for ordered membrane domains. These ordered membranes are then extracted from the TGN to form carrier vesicles rich in raft lipids and proteins, which are delivered as a bundle to the PM. Evidence supporting this raft-mediated mechanism is that cholesterol extraction or impaired sphingolipid synthesis reduces apical transport in epithelial cells (Hansen et al., 2000; Lipardi et al., 2000). Direct evidence of raft involvement in Golgi-mediated PM sorting has also been obtained by isolating a specific class of PM-directed vesicles in yeast. Vesicles marked by a raft-associated protein were enriched in sphingolipid and ergosterol and had more tightly packed membranes than membranes of the Golgi or parallel carrier vesicles (Klemm et al., 2009; Surma et al., 2011). In addition to their ability to sort lipids and proteins, raft domains may facilitate the budding of the vesicles due to the accumulation of line tension at the phase boundary between the raft and non-raft regions (Vind-Kezunovic et al., 2008; Surma et al., 2012).

Raft-based sorting in membrane traffic relies on proteins’ preferential partitioning to ordered membrane domains. This partitioning relies on biophysical differences between membrane domains: raft domains are relatively thick and tightly packed, whereas more disordered domains are thinner and less packed. Thus, thicker raft bilayers favor the incorporation of proteins with long TMDs (Diaz-Rohrer et al., 2014b; Lorent and Levental, 2015). Consistently, bioinformatic analysis of single-pass transmembrane proteins revealed that PM proteins generally have longer hydrophobic sections compared with Golgi resided proteins (Sharpe et al., 2010). Experimentally, shortening the length of the PM-resident transmembrane helix leads to intracellular retention of proteins (Munro, 1995; Diaz-Rohrer et al., 2014b). Together, these observations reveal that the length of a protein’s TMD is an important factor for cargo sorting at the TGN (Dukhovny et al., 2009). Similarly, other factors that determine a protein’s association with raft domains (TMD surface area, palmitoylation; Levental et al., 2010b) are also correlated with PM localization (Lorent et al., 2017). A recent finding shows that soluble protein cargo lipoprotein lipase can also be sorted into sphingomyelin-rich secretory vesicles through binding to an integral membrane protein Syndecan-1, which depends on its TMD length to target sphingomyelin-rich domains (Sundberg et al., 2019). Finally, we recently observed that the biophysical asymmetry of the PM might be a determinant of protein sorting, with structurally asymmetric TMDs accumulating at the PM (Lorent et al., 2020). This observation suggests the possibility of “asymmetric domains” in the Golgi membrane that warrant future investigations. Notably, raft domains in the Golgi have yet to be directly observed and characterized, with the size and morphology of this organelle hindering unambiguous observations.

## Shuttling Through the Endo-Lysosomal System

Endosomes and lysosomes organize the sorting, recycling, and degradation of PM membrane components internalized via endocytosis. Endocytosis involves numerous mechanistically parallel pathways, several of which have been associated with raft domains, including caveolae (Parton and Richards, 2003), clathrin-independent carriers (Kirkham and Parton, 2005), and GPI-enriched endocytic compartments (Sabharanjak et al., 2002). After endocytosis, these vesicles coalesce together and fuse with preexisting early endosomes, which are likely rich in cholesterol and sphingomyelin because of their origin at the PM. As these vesicles mature into late endosomes and lysosomes, their abundance of cholesterol and sphingolipids is reduced (Tharkeshwar et al., 2017), although evidence from detergent resistance and electron microscopy suggests that microdomains are still present in late endosomes (Sobo et al., 2007) and lysosomes (Simons and Gruenberg, 2000). One hypothesis for the depletion of cholesterol and sphingolipids in later stages of the endocytic pathway is that the raft components are recycled back to PM. Consistent with this notion, raft lipids are indeed enriched in recycling compartments and intermediates (Gagescu et al., 2000; Diaz-Rohrer et al., 2014a). Similarly, TMD constructs with high raft affinity localize at the PM, whereas mutations that reduce raft affinity lead to accumulation in late endosomes and lysosomes (Diaz-Rohrer et al., 2014b). Interestingly, it was recently suggested that cholesterol might be transferred from endosomes to phagosomes, in a membrane microdomain-dependent process of phagosome maturation (Rai et al., 2016). These ordered membrane domains in phagosomes act as a platform to recruit a team of dynein molecules, microtubule motors that drive phagosomes toward lysosomes for degradation.

Perhaps because of the unique lipid composition of the lysosome, some of the clearest microscopic evidence for raft domains in living cells comes from studies of the vacuole in yeast, which is analogous to the mammalian lysosome. While in the logarithmic phase, the yeast vacuole appears microscopically homogenous. In striking contrast, vacuoles of yeast grown to stationary phase show micron-sized coexisting liquid domains with distinct ergosterol abundances (Toulmay and Prinz, 2013). The depletion of ergosterol abolishes the formation of these domains, suggesting that they are sterol-dependent (Toulmay and Prinz, 2013; Tsuji et al., 2017). Later observations confirmed that these domains bear key biophysical hallmarks of ordered–disordered liquid phase separation, including domain coalescence and temperature-dependent, reversible demixing (Rayermann et al., 2017). The functions of these coexisting domains are not completely understood, although they may be involved in nutrient signaling or lipid transport at interorganellar contact sites (Murley et al., 2017), as discussed above. Finally, as domains can promote membrane bending (Hamada et al., 2007), phase separation could facilitate lipophagy through inward budding after binding to LDs (Seo et al., 2017). Thus, direct observations reveal large, ordered domains in live cells. The broad analogy between yeast vacuoles and mammalian lysosomes suggests that large-scale lipid phase separation may also be possible in mammalian cells under some conditions.

## Structure in the Fat–Lipid Phases in Lipid Droplets

Lipid droplets are often misconceived as structureless and relatively inert fat particles, when in fact, these organelles are vital for cellular homeostasis via energy storage and detoxification of free fatty acids. Although not directly related to ordered membrane domains, LDs present some fascinating biophysical features worth brief consideration. Droplets are typically spherical intracellular organelles 0.1–100 μm in diameter. Their major structural feature is a large core of neutral lipids (primarily triglycerides, but also cholesterol esters) surrounded by a phospholipid monolayer derived from the ER (Thiam et al., 2013). LDs are generated by a gradual accumulation of neutral lipids in the hydrophobic core of the ER membrane, until a critical threshold is exceeded, causing a dilation of the hydrophobic core into a “lens domain” (Chapman et al., 2019). This lens expands until the droplet buds off the ER facilitated by fat-inducing transmembrane proteins 1 and 2. Recent observations revealed that under certain metabolic scenarios, the core of LD transitions from a disordered liquid to a smectic liquid-crystal with clear long-range order observable by cryogenic electron microscopy (Mahamid et al., 2019). These experimental observations can be well described by a phase diagram that predicts a phase transition between liquid and liquid-crystal phases under near-physiological conditions due to the presence of cholesterol esters (Shimobayashi and Ohsaki, 2019). The crystallization of the droplet core due to excess sterol esters may have pathogenic significance in the case of non-alcoholic steatohepatitis (Ioannou et al., 2019) and atherosclerosis (Lada et al., 2002). Interestingly, in analogy to LDs, low-density lipoprotein particles were also reported to show reversible liquid crystalline phase transitions at varying temperatures and lipid compositions (Deckelbaum et al., 1977; Sherman et al., 2003).

## Function of Lipid Domains in Viral Assembly

Enveloped viruses, including important pathogens like human immunodeficiency virus (HIV), influenza, Ebola, and coronaviruses, are encapsulated by a lipid membrane originating from the membranes of host cells during virus budding. In many of these cases, the viruses bud from the PM with an envelope relatively rich in cholesterol and sphingolipids, suggesting the possibility of raft involvement in membrane assembly (Ono and Freed, 2001). Indeed, the PMs of HIV and influenza are highly enriched in raft-associated lipids, even compared with the PM from which they bud (Brugger et al., 2006; Gerl et al., 2012), indicating that these viruses bud from raft-like regions of the PM, and thus, raft domains may exist in virus membrane envelope.

The involvement of raft domains in HIV assembly and budding was recently supported by an exciting set of microscopic studies relying on validated lipid and protein probes for membrane domains (Podkalicka and Bassereau, 2019; Sengupta et al., 2019). The nascent viral buds specifically recruited raft lipid probes like fluorescent cholesterol and sphingomyelin and lipidated proteins, while excluding markers of disordered domains (Sengupta et al., 2019). The observations also suggested that rather than budding at preexisting raft domains on PM, ordered domains were recruited and assembled actively after binding the HIV matrix protein Gag to the mammalian PM, consistent with previous studies (Ono and Freed, 2001). Gag is known to interact with the PM via binding to the charged phosphoinositide lipids PIP2 (Ono et al., 2004), which may couple with sphingolipid-rich outer leaflet (Lorent et al., 2020) to produce a transbilayer ordered domain. Similar coupling behavior has also been inferred between inner leaflet PS with long saturated acyl chains and outer leaflet GPI-anchored proteins (Raghupathy et al., 2015). Considering PS is also enriched in the HIV membrane (Lorizate et al., 2013), it is possible that enrichment of PS at the assembly site could further enhance coupling between the two leaflets to facilitate domain formation. Furthermore, the myristoyl chain in Gag protein may enhance its interaction with PIP2 and PS with saturated chains and stabilize raft domains.

The precise roles of membrane domains in viral function have not been completely resolved; however, several possibilities have been suggested by experimental evidence. First, similar to protein sorting in eukaryote membrane traffic, rafts may function as sorting platforms for selective incorporation/exclusion of viral proteins. Consistent with this notion, many viral envelope proteins are posttranslationally palmitoylated, including those of influenza (Kordyukova et al., 2008; Engel et al., 2010), HIV (Bhattacharya et al., 2004), the severe acute respiratory syndrome-related coronavirus (Petit et al., 2007), and Chikungunya (Zhang et al., 2019). Such palmitoylation would help target these viral proteins to PM rafts, as posttranslational palmitoylation has been established as a crucial signal for the recruitment of proteins to ordered domains (Levental et al., 2010b). It has been demonstrated that the ordered environment of the viral membrane stabilizes and affects the conformation of some envelope glycoproteins (Salimi et al., 2020), suggesting that rafts can both recruit and regulate viral protein function. Another important possibility is that the presence of a large membrane domain could promote the outward curvature required for the generation of a viral bud (Chazal and Gerlier, 2003). Moreover, the unique lipid composition of viral envelopes may promote their stability in cold air (Polozov et al., 2008), facilitating transmission. Consistently, the extraction of cholesterol from HIV and other viruses inactivated viral infectivity (Graham et al., 2003). Finally, raft domains on the viral envelope may play an essential role in the membrane fusion required for viral entry into infected cells. It was observed that the HIV fusion protein gp41 preferentially attaches to the interface between ordered and disordered domains on the host membrane, perhaps due to the line energy present at this interface. The boundaries between the raft and non-raft domains on both the viral and host membranes are energetically costly, and this energy could be reduced by membrane fusion, potentially providing an energetic driver for viral entry (Yang et al., 2016, 2017).

## Pulmonary Surfactant

Lung surfactant is a thin film of lipids and proteins at the surface of alveoli secreted by type II alveolar epithelial cells. The main function of LS is to reduce the surface tension at the air–alveoli interface and thereby protect alveoli from collapse during the rapid area compression–expansion involved in the breathing cycle (Perez-Gil, 2008). Surfactant deficiency, e.g., in premature neonates, can result in neonatal respiratory distress syndrome, which can be treated by administrating exogenous surfactant, consisting of lamella-forming mixtures of phospholipids, cholesterol, and proteins (Andersson et al., 2017). Lipids account for ∼90% of LS dry weight, with the most abundant being dipalmitoylphosphatidylcholine (40–70%) and cholesterol (10%) (Veldhuizen et al., 1998). The composite structures (lipid monolayer at the air/liquid interface, overlying multiple bilayers; Follows et al., 2007) and phase behaviors of LS are believed to be critical for its function. Neutron scattering experiments revealed multilamellar stacks that appear to be in the disordered phase at physiological temperature (Follows et al., 2007). This is somewhat surprising considering the high concentration of fully saturated lipids may be expected to form gel phases below 40°C. Although a membrane composed solely of pure So phase can efficiently reduce surface tension (Veldhuizen et al., 1998), it would not be as effective in the dynamic environment, experiencing rapid changes in shape and area. Thus, other unsaturated phospholipids and cholesterol are included in LS to provide essential fluidity and flexibility. These mixtures tend to form a variety of structures, including coexisting ordered and disordered domains (Casals and Canadas, 2012). This phase separation may facilitate the bending of LS films to provide membrane reservoirs for compression–expansion during respiration (Casals and Canadas, 2012). Moreover, various LS proteins partition differently into these lipid domains, or the domain interface, to stabilize the complex LS structures (Canadas et al., 2020). Ultimately, although the exact structure and dynamical properties of native LS are not yet fully elucidated, it is likely that lipid domains play an important role in maintaining key biophysical properties. Whether such domains may also play roles in molecular signaling, gas exchange, or other LS processes should also be explored.

## Milk Fat Globule Membrane

Mammalian milk is rich in lipids packaged in the form of fat globules surrounded by a milk fat globule membrane (MFGM). The MFGM is an unusual tri-layer, whose inner monolayer is comprised of polar phospholipids and proteins surrounding a core of neutral triglycerides. This typical LD-like configuration is then surrounded by an outer bilayer, presumably acquired during extrusion of the LD-like fat globule through the apical surface of mammary epithelial cells (Lopez et al., 2010). The specific lipid composition of this unusual membrane differs somewhat among various mammalian species; however, it is broadly composed of approximately equimolar PE, PC, and sphingomyelin, with a relatively high abundance of cholesterol (chol/SM ∼ 3) (Et-Thakafy et al., 2017). Consistent with this composition and its origin in the apical PM of epithelial cells, the MFGM separates into coexisting ordered and disordered membrane domains over a large temperature range that includes physiologically relevant temperatures (Lopez et al., 2010; Et-Thakafy et al., 2017). Atomic force microscopy showed that these domains were different by approximately ∼1 nm in height (Murthy et al., 2016), consistent with the expected differences between ordered and disordered phases (Heberle et al., 2020). Domains with morphology reminiscent of both liquid (circular) and solid (jagged and irregular) phases are observable under various conditions, so it is not yet clear what determines the precise nature of the ordered phase. The physiological purpose of this phase coexistence is also not understood. It has been suggested that milk components can be antimicrobial, protecting against food-borne antigens. In this context, it is possible that the phase-separated MFGM could act as a “sink” for an unproductive fusion of viruses (Yang et al., 2016).

## Conclusion and Outlook

The presence of lipid domains on non-PM membrane systems is fully consistent with, and a logical extension of, the lipid raft hypothesis. The evidence above combines to strongly support the relevance of lipid domains in various biological membranes, highlighting a general principle of membrane compartmentalization regulating various biological processes. The underlying biophysical insights generated from the intensive interest in PM lipid rafts are equally applicable to non-PM membrane systems. The experimental challenges of studying intracellular membranes have been a formidable barrier to a detailed understanding of their structure and organization: such membranes are not accessible to external labels or manipulations; organelles are often small and have a variety of complex shapes (tubules, folds, etc.); and various membranes are tightly packed inside the cells making unambiguous identification difficult. However, several methods have been recently developed that allow observations of large-scale domains, such as enlarging organelles through hypotonic swelling or fatty acid feeding (Shen et al., 2017; King et al., 2020). In parallel, the continuing advances in microscopic and spectroscopic technologies, and novel membrane sensitive probes (Klymchenko, 2017), are pushing toward the necessary temporal and spatial resolutions for unambiguous detection of membrane domains (Stone and Veatch, 2015; Sezgin et al., 2017, 2019; Stone et al., 2017). Novel technologies such as label-free Raman microscopy (Shen et al., 2017) and cryoEM imaging of membrane properties (Heberle et al., 2020) are opening doors to dissect membrane domains with minimal perturbations inaccessible with typical fluorescence microscopy. Finally and most importantly, lateral heterogeneity is likely a widespread phenomenon across various biological membranes, prompting more directed attention to non-PM systems that have the potential to extend our understanding of the structure and function of living membranes.

## Author Contributions

All authors listed have made a substantial, direct and intellectual contribution to the work, and approved it for publication.

## Conflict of Interest

The authors declare that the research was conducted in the absence of any commercial or financial relationships that could be construed as a potential conflict of interest.
